# Mothers’ Subjective Well-Being after Having a Second Child in Current China: A Case Study of Xi’an City

**DOI:** 10.3390/ijerph16203823

**Published:** 2019-10-10

**Authors:** Jianghua Liu, Zhongliang Zhou

**Affiliations:** 1 The Institute for Population & Development Studies, Xi’an Jiaotong University, Xi’an 710049, China; 2 The School of Public Policy & Administration, Xi’an Jiaotong University, Xi’an 710049, China; zzliang1981@xjtu.edu.cn

**Keywords:** life satisfaction, children’s well-being, low fertility, two-child policy, son preference, fertility-friendly policy, family intervention

## Abstract

The introduction of the two-child family planning policy in China calls for a study of the response of mothers’ subjective well-being after the birth of a second child. Generally focusing on Western countries, previous studies suggested that a series of factors could influence the response, but insufficient attention has been paid to the relative importance of these factors so far. Based on survey data from mothers of two children in the Xi’an metropolitan area, Shaanxi Province, China, our study indicates that the important factors associated with mothers’ life satisfaction after having a second child were, in general, common to Western countries and China. There were also two factors somewhat unique to China: positive adjustment (i.e., becoming happier) by firstborn children (average age, 6 years old) following a sibling’s birth, predicted enhanced life satisfaction for mothers; additionally, mothers who had both a son and a daughter reported the highest increase in life satisfaction, while mothers who had two sons reported the lowest increase. Socioenvironmental constraints (i.e., parenting pressure and work–family conflict) had a larger association with mothers’ life satisfaction than individual ideational factors (e.g., family orientation and fertility desire). These findings suggest that fertility-friendly policies and convenient family intervention institutions are needed to alleviate potential undesirable consequences and improve maternal life quality following a second childbirth so that the two-child policy can be a success.

## 1. Introduction

The introduction of the two-child family planning policy since the end of 2013, initially selectively and then universally, has marked a major social transition in China; for decades before that time, almost all urban couples and some rural couples were not allowed to have a second child after having their first child in the country [1,2]. It was expected by the Chinese central government that the people would welcome the new policy; family size that had been, on average, far below replacement level (i.e., <2) would improve; and having a second child would enhance family happiness, a major criterion in judging governance success in current China [2,3,4] (for the discussion about American society, see ref. [5]). Consequently, it is warranted to study parental subjective well-being in response to a second childbirth, detect important factors that constrain people’s well-being and design practical measures to alleviate such constraints, which is a challenge confronted, to varying degrees, by other low-fertility countries [6,7].

Most of the previous studies in this area focused on Western countries and the cognitive component of subjective well-being in terms of life satisfaction, the nature of which lies in the gap between ideal and actual life quality [8,9,10,11]. It is sometimes argued that one’s well-being exists on a treadmill and life events cannot change it for a long time, i.e., the adaptation or set-point theory of happiness [5,7]. Some psychological studies suggest that this argument might apply more to the affective components of subjective well-being (e.g., positive versus negative moods and emotions), which are more attributed to personality and other genetically determined traits [8]. A series of studies indicate that as a major transition in one’s life, childbearing could have long-lasting effects on cognitive well-being, and such an effect can be modified by various individual traits, e.g., life course, gender, and marital status [12,13,14]. Recent studies also indicate the significant effects of institutional factors—social security, childcare system, work–family conflict under given institutions—and individual attitudes toward the relationship between work and family, i.e., family orientation, on one’s life satisfaction after childbearing [9,10,15,16,17]. Perceived psychological and financial stresses play a key role in linking institutional factors at one end and life satisfaction at the other [6]. Sometimes such stresses affect life satisfaction indirectly by activating the depression component of neuroticism, the major personality trait affecting cognitive well-being through the mediation of affective well-being [8].

Besides the above factors, the following factors could also be relevant to one’s life satisfaction after having a second child, as predicted from the perspective of a mismatch between ideal and actual quality of life. First, as an individual ideational factor, fertility preference before a second child may be an important influencing factor. Fertility desire/intention can be seen as a desire/goal with respect to family size, and thus having a second child or not can be seen as fulfilling the desire/goal or not. By contrast, any unintended pregnancy can be seen as contradictory to one’s fertility preference, and thus will have a negative effect on life satisfaction [18,19]. It is worth noting that fertility desire and intention might have independent influences on post-second birth well-being [20]. In such societies like China and other East Asian regions, son preference is an important aspect of fertility preference [21], and thus the sex composition of two children may also exert an influence on one’s subjective well-being following the second birth.

Second, family members, including the firstborn child or the mother’s partner, could influence the mother’s well-being. Parent–offspring conflict theory in evolutionary biology suggests that there might be a conflict between parents and children over having another child [22,23,24]. If the firstborn child supports his/her parents to have a second child and becomes happier after seeing the sibling, parents may also feel happier [8,11]. By contrast, parents may become less happy if the firstborn child experiences negative moods after a sibling’s birth; such negative moods are expected among some, if not all subgroups of firstborns [25,26]. This prediction especially makes sense for those countries where families have become child-centered; for example, in China, after decades of the one-child policy, an only child’s needs take priority over those of other family members [27,28]. In the same way, it can be predicted that a partner’s emotional support for having a second child will increase the mother’s happiness, as harmony with a partner can be a major source of happiness [29]. Practical support might be another important influencing factor [26,30]. For example, if a husband increases his share of housework after the second birth, his wife’s housework load would be reduced. Consequently, overall life satisfaction would not change much or might even improve after having the second child. This mechanism can be seen as similar to social institutions adapting to women’s aspiration to work [6,9].

In this study, we analyze the influence of the factors mentioned above on Chinese mothers’ life satisfaction following the birth of a second child. Furthermore, we compare the relative importance or effect size of individual factors; insufficient attention has been explicitly given to the topic, but the existing literature suggests that these factors might not be equally important to maternal well-being. China differs somewhat from Western countries in its social and cultural background (e.g., the above-mentioned child-centered family culture that evolved in the context of the one-child policy and low gender equity in the division of domestic labor [31]). Thus, our study helps to reveal common (to Western low-fertility countries) and unique factors influencing mothers’ subjective well-being after the birth of a second child in China, detect the most important happiness-modifying factors, and propose measures targeting the improvement of maternal well-being so that the two-child policy can be a success.

## 2. Materials and Methods

### 2.1. Background of the Survey

From the early 1980s to the early 2010s, the one-child policy was implemented in China. According to the policy, almost all couples in urban areas were allowed to have only one child [1,32]; in rural areas, the one-child policy was implemented less strictly (e.g., in most provinces, the rural couples whose firstborn child was a girl were allowed to have a second child). After three decades of the one-child policy, the fertility level was far below replacement level, the signs of shortage of labor force had appeared, and the Chinese population was increasingly aging, which all threatened the sustainable socio-economic development of the country [2]. Thus, the Central Committee of the Communist Party of China (CPC) made a decision to introduce the selective two-child family planning policy on 12 November 2013. According to the new policy, couples where either the husband or wife was an only child (only-child couples) were allowed to have two children.

In October 2015, the government of Xi’an, a metropolitan city in Western China, requested a research team, with the first author as the principal investigator, to investigate the situation of implementing the policy and whether one of its major goals was fulfilled: happiness of couples with one child improved after they had their second child [2]. The survey area displayed a pattern of very low fertility [33]; actually, even after the introduction of the universal two-child policy since 2016, i.e., every couple was allowed to have two children, the total fertility rate was below 1.5 according to the 2017 China Fertility Survey [34].

All subjects gave their informed consent for inclusion before participating in the survey. The survey was conducted in accordance with the Declaration of Helsinki and was approved by the Biomedical Ethics Committee of Xi’an Jiaotong University (No. 2015-636).

### 2.2. Survey Data

Since the first cohort of second-born children under the selective two-child policy were born around September 2014, we sampled all women in only-child couples (the total was around 2500) who gave birth to their second child within one year from that time, i.e., September 2014 to August 2015. They were distributed across all 15 districts/counties (the first level of administrative division) of the Xi’an metropolitan area. The effective response rate was relatively low: most of them could not be contacted or failed to contribute to an effective questionnaire, due to issues with feeding, childcare, conflict with work time or relocation. Finally, 596 effective questionnaires were successfully collected through either a telephone or self-administered approach. Studies based on data across the whole country indicated that only-child couples and other couples did not differ significantly in desire and intention to have a second child, and life satisfaction following a second childbirth [35,36]. Thus, although the sample focused on women in only-child couples and cannot perfectly represent the population in the area, it helped to reveal the general pattern of the response of mothers’ subjective well-being to a second childbirth and how a series of factors influenced the response.

In the questionnaire, the key question asked was: “Compared to the time before having the second child, how do you think your current life is?” Available options were: “much less satisfied”, “somewhat less satisfied”, “the same satisfied as before”, “somewhat more satisfied” and “much more satisfied”. Our indicator of subjective well-being after a second childbirth was borrowed from a measure of well-being in the British Household Panel Survey (e.g., “more happy than usual”; see ref. [14]) and a measure of sense of gain in the Chinese Social Survey (i.e., whether one’s living standard improved compared to five years ago according to one’s own evaluation; see ref. [37]).

Besides the key question, the following six groups of questions were asked: (1) Background information: family’s annual income, wife’s and husband’s occupation and education, wife’s age, time since the second birth, the firstborn child’s age at time of survey, and family settlement (main city vs. inner suburb vs. outer suburb). (2) Postnatal depression in terms of whether the mother felt depressed during the first month after the second childbirth (if the answer was yes, it was validated by further inquiring into underlying reasons, such as insufficient support from family members). (3) Fertility preference: fertility desire before the second birth (treated as a continuous variable and measured by the mean score of desire to have a second child before and during pregnancy with the second child, with both types of desire measured using a 5-point scale from “very willing” to “not willing at all”); the occurrence of an unintended pregnancy (a pregnancy was classified as mistimed if “unintended pregnancy” was chosen as a reason for “having the second child during the recent two years” but not for “having the second child”, as unwanted if “unintended pregnancy” was chosen for both questions, and planned if “unintended pregnancy” was chosen for none of the questions); and the sex composition of two children. (4) Responses of the husband and the firstborn child to having a second child (emotional support—when an unsupportive attitude was mentioned, it was further validated by inquiring for underlying reasons—for having a second child during pregnancy with the child, whether the firstborn became happier after the birth of a sibling, and changes in the husband’s share of housework before and after the second childbirth). (5) Factors constraining parenting (work–family conflict, pressure of parenting two children). (6) Family orientation in terms of whether the mother was willing to sacrifice career development for parenting two children. Table 1 lists the descriptive statistics of all variables.

### 2.3. Statistical Methods

In the statistical model analyzing the influence of factors mentioned above on mothers’ subjective well-being, the response variable was the mother’s life satisfaction after the second childbirth, an ordinal variable with three levels, coded as 1: “less satisfied”; 2: “the same satisfied as before”; and 3: “more satisfied” (to lessen the problem of reporting bias (e.g., ref. [38]), we collapsed the original 5-point measure into a 3-point one). All other variables were included as predictors in the model, which constituted the base model in the study. Given that the sample was of a medium size, we focused on an ordered logistic regression model, which is more parsimonious than a multinomial model, using the statistical package “ordinal” run on *R* [38,39,40,41]. The original model was a proportional odds one: $\mathrm{logit}\left[P\left(Y\leq j\right)\right]=\theta_{j}-\sum \beta_{i}x_{i}+\varepsilon$. Here, *j* took values of 1, 2, ···, *J* − 1 (*J* was the number of categories of ordinal response variables) and $P\left(Y\leq j\right)$ was the cumulative probability of occurrence of *Y* up to *j*. In this study, *J* = 3; thus, the base model actually consisted of two logistic regression models, each with a *j*-specific intercept *θ_j_* and a set of *β_i_* (i.e., the regression coefficient of the *i*th regressor). Since there was a minus sign after *θ_j_*, a positive *β_i_* implied a lower likelihood of having decreased life satisfaction after the birth of the second child. A model check indicated that the proportional odds assumption was violated for some predictors, i.e., *β_i_* varied with *j*, so we adapted the above model accordingly to get a partial proportional odds model (here, the coefficients of predictors satisfying the proportional odds assumption were coded as *β*, but the coefficients that did not satisfy the assumption were coded as *θ*). For example, a regressor might be related to a lower likelihood of feeling less satisfied after having a second child, but not necessarily to a higher likelihood of feeling more satisfied—i.e., 1 − likelihood of experiencing reduced or invariant satisfaction—or vice versa. The following proverb helps to understand the phenomenon: “Money cannot buy happiness, but buy off unhappiness.”.

The relative importance of the given predictors was assessed with a likelihood-based method [41,42,43]: (1) a simplified model was obtained by excluding the predictor (or group of predictors) in question from the base model including all relevant predictors (see above); (2) the difference in the corrected Akaike Information Criterion or AIC values of the two models (ΔAIC_c_ = AIC_c,simplified_ − AIC_c,base_) was used to assess the relative importance of the predictors. If ΔAIC_c_ was negative, the excluded predictor was not important, as excluding it from the base model led to a smaller distance between the model and reality; by contrast, if ΔAIC_c_ was positive, the predictor was considered as important. The larger the ΔAIC_c_ value, the more important the predictor in predicting the response variable.

## 3. Results

We first report the main descriptive statistics of variables used in the partial ordered logistic regression analysis of life satisfaction after having a second child. Compared to the time before, about 19.5% of mothers felt less life satisfaction after having the child, 48.7% felt the same as before, and 31.9% felt more satisfied. On average, the mothers were 32.8 years old, their first child was 6.3 years old, and their second child was 8.9 months old at the time of the survey. Note that due to the restriction of the previous one-child policy, the interbirth interval was evidently longer than the natural modal pattern of 2–3 years, a phenomenon presumably unique to current China. The majority of mothers came from the main or inner city of the metropolitan area, 17% were from the inner suburbs, and 12% were from the outer suburbs (note: the urbanization rate in the area was about 72% at the time of the survey). Three-quarters of the surveyed mothers or their husbands had finished higher/tertiary education. Most second births resulted from family planning, 7.9% from mistimed pregnancy, and 19.3% from unwanted pregnancy. Note that the study only investigated mothers whose pregnancy resulted in a live second birth, and the unintended pregnancy rate could be higher if all pregnancy outcomes are considered, including live births, abortions, stillbirths, etc. About 20% of mothers felt depressed within the first month after the second birth. During the pregnancy, a minor proportion of husbands and firstborn children did not show an explicitly supportive attitude about having a second child. Close to 57% of firstborns became happier after seeing their sibling. For about 30% of mothers, the husband improved his share of housework after the birth of the second child, but for others, such an improvement did not happen. Three-quarters of mothers felt moderate or severe conflict between work and parenting two children, and about 71% felt moderate or strong pressure in parenting two children. Finally, 22% of mothers were very willing to sacrifice their career development to parent two children, 35% were only moderately willing to do so, and 18% were reluctant to do so; other mothers said they were not professional women and thus this question was not applicable to them.

Table 2 shows the estimates from the partial ordered logistic regression analysis of mothers’ self-evaluated change in life satisfaction after having a second child. Life satisfaction was correctly classified for about 61% of mothers by the base model.

The factors that significantly influenced post-second birth life satisfaction were as follows: (1) Background factors. Older age predicted a higher likelihood of decreased life satisfaction (odds ratio (OR) = 1.09, 95% confidence interval (CI) = 1.00, 1.19; Table 2), but it was not related to the likelihood of experiencing enhanced life satisfaction (OR = 1/0.995 ≈ 1.01, 95% CI = 0.93, 1.09); evidently, the likelihood of experiencing the same life satisfaction as before decreased with age. (2) Postnatal depression. Feeling depressed during the first month after the second childbirth did not predict the likelihood of enhanced life satisfaction (OR = 0.96, 95% CI = 0.59, 1.57), but it was associated with a higher likelihood of decreased life satisfaction (OR = 1/0.428 ≈ 2.34, 95% CI = 1.39, 3.92); thus, experiencing depression meant a lower likelihood of feeling the same satisfaction as before.

All three indicators of fertility preference were significantly related to life satisfaction after the second birth. First, a weaker desire to have a second child before the birth was negatively associated with the likelihood of enhanced life satisfaction after the birth (OR = 0.67, 95% CI = 0.52, 0.85). Second, mothers who had a second child due to an unintended pregnancy had more difficulty feeling increased life satisfaction than those whose second child was a result of family planning (mistimed pregnancy: OR = 0.85, 95% CI = 0.45, 1.61; unwanted pregnancy: OR = 0.61, 95% CI = 0.38, 0.96). Third, compared to those with two sons, mothers with other gender combinations of children were more likely to feel more satisfied after the second birth: first child a son and second a daughter, OR = 2.22 (95% CI = 1.39, 3.53); first child a daughter and second a son, OR = 1.72 (95% CI = 1.09, 2.74); both daughters, OR = 1.53 (95% CI = 0.94, 2.50).

Responses of family members to the second child constituted another group of significant influencing factors. Compared to the case where the firstborn child became happier after the birth of a sibling, the case where the level of happiness was the same predicted a lower likelihood of enhanced life satisfaction (OR = 1/1.791 ≈ 0.56, 95% CI = 0.36, 0.88), and the case where the firstborn child became less happy predicted a higher likelihood of decreased life satisfaction for the mother (OR = 2.26, 95% CI = 1.13, 4.52). The firstborn’s emotional support for the mother during the pregnancy with the second child was not significantly associated with the likelihood of enhanced life satisfaction of the mother after the birth (Table 2). It is worth noting that the effects of the firstborn child on the mother’s happiness did not vary significantly with the child’s age (interaction between age and these factors: $\chi_{4}^{2}=4.24$, *p* = 0.38). The husband’s emotional support during the pregnancy was positively associated with the likelihood of enhanced life satisfaction for the mother after the birth (OR = 1/0.696 ≈ 1.44, 95% CI = 0.96, 2.14). Compared to the case where the husband increased his share of housework after the second birth, there was a lower likelihood of feeling more satisfied if the husband’s share did not increase (OR = 1/1.783 ≈ 0.56, 95% CI = 0.37, 0.84).

Both constraining factors had a major effect on life satisfaction after the second birth. Compared to the case where having a second child did not have a negative influence on career development, a moderately negative effect would bring a lower likelihood of enhanced life satisfaction after the birth (OR = 1/2.547 ≈ 0.39, 95% CI = 0.23, 0.66). If having a second child had a strong negative influence on career development, the likelihood of feeling less satisfied with life would be enhanced (OR = 2.56, 95% CI = 1.26, 5.24), and the likelihood of feeling more satisfied declined after the second birth (OR = 1/1.947 ≈ 0.51, 95% CI = 0.29, 0.90). Compared to the case of feeling no pressure in parenting two children, feeling strong pressure meant a higher likelihood of decreased life satisfaction (OR = 3.89, 95% CI = 2.03, 7.47), but such pressure did not significantly predict the likelihood of enhanced life satisfaction (OR = 1/0.993 ≈ 1.01, 95% CI = 0.62, 1.65); evidently, strong pressure was related to a lower likelihood of feeling the same satisfaction as before having the second child.

Lastly, compared to mothers who were very willing to sacrifice their career development to parent two children, other mothers would have a higher likelihood of feeling less satisfied and a lower likelihood of feeling more satisfied after the second birth (moderately willing, OR = 0.41, 95% CI = 0.25, 0.66; reluctant, OR = 0.35, 95% CI = 0.20, 0.62; nonworking mothers, OR = 0.52, 95% CI = 0.30, 0.89).

Likelihood analysis indicated that removing two socioenvironmental constraints on parenting children—perceived parenting pressure and work–family conflict—caused the largest increase in AIC_c_ (36.09), indicating that these were the most important factors related to the mother’s life satisfaction. Other important groups of modifying factors included fertility preference (desire to have a second child before birth of the child, whether the second childbirth resulted from an unintended pregnancy, and the sex composition of the two children; ΔAIC_c_ = 17.56), the mother’s family orientation (ΔAIC_c_ = 10.25; Table 2), the husband’s emotional and practical support (ΔAIC_c_ = 8.32), feeling depressed during the first month after the second childbirth (ΔAIC_c_ = 6.16), and the firstborn’s happiness (ΔAIC_c_ = 3.51). This sequence of ΔAIC_c_ corresponds to the relative importance of these groups of factors in modifying the mother’s life satisfaction after a second birth.

## 4. Discussion

### 4.1. Interpretation and Implications

Based on survey data from Xi’an City, China, we conduct a relatively comprehensive investigation of the impact of individual, familial and social factors on mothers’ subjective well-being after a second birth, which is timely because the two-child policy has been in effect fairly recently. Our study indicates that the well-studied happiness modifiers in Western countries—e.g., parenting pressure, work–family conflict, family orientation, and partner’s emotional and practical supports—were also significant for Chinese mothers after having a second child.

Furthermore, the current study provides one of the first evaluations of the relative importance of these factors: post-second birth life satisfaction was more subjected to socioenvironmental constraining factors than to individual ideational factors, a finding consistent with previous studies on affective vs. cognitive subjective well-being [8,44]. Through its influence on the likelihood of experiencing decreased life satisfaction, parenting pressure was the paramount factor modifying post-second birth well-being. This finding is consistent with the results from European countries as well as the current popular view in China: parenting children is so hard that the government should design suitable family policies to ease parenting [6,16,45]. An interesting observation might be worth noting: a previous study conducted in the same area indicated that expected parenting pressure was less important than one’s childbearing ideology in predicting the desire and intention to have a second child among one-child mothers [43]. Thus, the relative importance of parenting pressure changed before (i.e., reproductive decision-making) and after (i.e., reproductive after-effects) the second birth. Although not as paramount as socioenvironmental constraints, ideational factors—fertility desire, family orientation, et al.—played also an important role in shaping post-birth life satisfaction. For example, the mother’s family orientation was the second most important single factor, and mothers with more traditional orientation were more likely to experience enhanced life satisfaction after having a second child (Table 2; see also ref. [10]).

This study also obtains important results regarding the influence of family members. First, consistent with our expectation, the firstborn child had an important influence on the mother’s life satisfaction after the second birth. Kohler and Mencarini [11] previously raised the following questions: Is parents’ subjective well-being influenced by children’s well-being? Is the relationship between them complementary or a trade-off? Evidently, this study indicates that the answer to the first question is yes and to the second question is basically complementary. Whether this is the case in other cultures warrants further research, but one thing is clear now: difficulties with adjusting to a sibling’s birth were also observed in firstborns in other countries [26]. Second, we find that the husband’s emotional support during the pregnancy with the second child helped to improve the mother’s life satisfaction after the birth, which is consistent with findings emphasizing the importance of social support in promoting maternal mental well-being [29,30]. However, the husband’s emotional support was less important than his practical support in terms of sharing housework (Table 2; see also ref. [46]).

Some factors did not show an expected influence on mothers’ subjective well-being; e.g., sex composition of children had an influence contrary to the expectation based on assumed son preference and firstborn’s emotional support during the pregnancy was not significant. Presumably, so-called son preference might have waned in the survey area and now is not as strong as we generally think; instead, both the current study and recent fertility surveys suggest that a mixed-sex preference may have truly become prevalent in China, just as in Western countries (e.g., refs. [34,47,48]). The work by Jin et al. [49] provided one clue, presumably unique to China, as to the transition in sex preference for children: in current China, there is a very high economic cost for men to marry in a context of a surplus of men and a shortage of marriageable women, which might make parents very reluctant to have more than one son, especially in rural areas. The strong practical burden of parenting sons also helps to explain the significant negative effect of having two sons on post-second birth life satisfaction, a result in contrast to that of Margolis and Myrskylä [47], who found little effect of sex preference on parental subjective well-being in European countries. As far as it concerns emotional support from the firstborn child, the work of coordinating family members when having a second child must have been done beforehand, because couples decided to give birth to the child; then, the conservative or disagreeable attitude of the firstborn child could change later and have no significance for maternal life satisfaction after the second childbirth.

This study has important implications for China and possibly also for other low-fertility countries. First, a substantial fraction of mothers, close to 20%, experienced a decrease in subjective well-being after having a second child. The figure for mothers in general might be similar (for the similarity of life satisfaction after a second birth between mothers in only-child couples and those in other couples, see ref. [36]). This finding suggests the need to set up family-friendly institutions in China to alleviate maternal parenting pressure and work–family conflict, in the light of their dominant effect in restraining people’s life satisfaction. Currently, there are no formal public childcare centers for children under three years old, and maternal leave provides little pay and is relatively short in China, which are major institutional challenges also confronted by families in some European countries [6,45,50]. Second, given that children’s happiness is an important factor influencing women’s life satisfaction, it is helpful to make family intervention services available and convenient, such as childcare training for mothers and psychological intervention for firstborn’s negative adjustment to the birth of a sibling. Family intervention is also expected to alleviate suffering from postnatal depression and negative effects of unwanted pregnancy (the prevalence rates of both were around 20% in this sample) (see refs. [20,29]). The above proposed measures should be welcomed by all parents, irrespective of their parity or gender, and the introduction of the two-child policy makes the supply of relevant services more imperative. By taking such measures, the Chinese government may get closer to its goal of helping one-child couples to feel happier if they manage to have two children [2], and the two-child norm may spread more quickly and widely as a result of social learning [14].

### 4.2. Limitations

Despite the contributions discussed above, limitations of the study should be mentioned. First of all, a retrospective research design made the measurement of some indicators suffer from potential reporting bias. For example, a mother who experienced decreased life satisfaction after having a second child more likely reported postnatal depression, work–family conflict, etc. A prospective study based on a longitudinal survey could help to fill the gap. Since the selective two-child policy was introduced for only one year at the time of the survey, it was difficult to collect follow-up data; however, a longitudinal study should and can be done in the near future to validate or invalidate our findings.

Additionally, there were a few potential sampling biases. First, the low effective response rate (~24%) implies that we might have missed mothers who were busy with their work or other responsibilities. Second, the proportion of mothers with a tertiary degree could have been over reported [33], which might be explained by the fact that only-child couples (either the husband or wife was an only child) were generally better educated. Third, almost a third of the mothers with two children were unemployed, which raises the question of whether unemployed mothers were overrepresented (for 2010 unemployment rates among reproductive-age women in the area, see ref. [33]). There were several causes of unemployment for mothers with two children: they came from economically well-off families, so they did not need to work for income, or they gave up their jobs because of work–family conflict despite needing money to parent two children, or they were fired because they could not focus on their jobs after the second childbirth. Presumably, each reason can hold for a subgroup of unemployed mothers; due to the low response rate, we cannot exclude the possibility that our sample did not well represent each subgroup. Given the above sampling biases, one should proceed with caution in generalizing the findings here to a larger population, and more surveys are warranted in other regions to better design fertility-friendly services.

Last but not least, we did not study the association of fertility and happiness in husbands, who may deal with different factors that reinforce or challenge their post-second birth well-being. Given that a goal of the two-child policy is to improve couples’ well-being after having a second child, it is warranted to fill the gap in the future.

## 5. Conclusions

The factors significantly influencing mothers’ subjective well-being were, in general, common to Western countries and China, such as parenting pressure, family orientation, work–family conflict, and housework division. However, two factors seemed to be unique to Chinese mothers: (1) due to restriction of the previous one-child policy, firstborn children were generally old enough at the time of the second childbirth to understand what was going on, and if they felt happier after a sibling’s birth, the mother tended to feel more satisfied with life; and (2) mothers with two sons had the lowest gain in life satisfaction, while those with a son and then a daughter had the highest gain. Socioenvironmental constraints had a larger effect on mothers’ life satisfaction than individual ideational factors. These findings suggest that fertility-friendly policies and family intervention institutions should be introduced to alleviate potential undesirable consequences following a second childbirth, e.g., the psychological pressure of parenting two children, firstborn’s negative adjustment, unwanted pregnancy, and postnatal depression (in the sample, the prevalence rate of each cannot be neglected). Such measures will help to ensure that maternal life quality tends to be improved after the birth of a second child and the two-child family planning policy is a success.

## Figures and Tables

**Table 1:** Descriptive statistics of variables used in statistical modelling.

Group of Variables	Variables	Statistics ^1^
Response variable	Mother’s life satisfaction after the second childbirth	
	Decreased	0.195
	Same	0.487
	Increased	0.319
Background factors	Mother’s occupation	
	Timewise inflexible ^2^	0.455
	Timewise flexible	0.154
	Housewife	0.334
	Others	0.057
	Husband’s occupation	
	Timewise inflexible	0.606
	Timewise flexible	0.267
	Jobless	0.022
	Others	0.106
	Mother’s education	
	Pre-college level	0.265
	College/university level	0.646
	Postgraduate level	0.089
	Husband’s education	
	Pre-college level	0.258
	College/university level	0.612
	Postgraduate level	0.129
	Family settlement	
	Main city	0.711
	Inner suburb	0.168
	Outer suburb	0.121
	Family income ^3^	
	Low	0.238
	Middle	0.485
	High	0.277
	Mother’s age (years)	32.83 (3.576)
	Firstborn child’s age (years)	6.327 (2.897)
	Life course since second childbirth (months)	8.860 (3.500)
Postnatal depression	Did you ever feel depressed during the first month after the birth of the second child?	
	No	0.795
	Yes	0.205
Fertility preference	Desire to have a second child before birth of the child	2.089 (0.798)
	How was the second birth planned?	
	Planned pregnancy	0.728
	Mistimed pregnancy	0.079
	Unwanted pregnancy	0.193
	Gender composition of two children	
	Son/son ^4^	0.295
	Son/daughter	0.245
	Daughter/son	0.258
	Daughter/daughter	0.201
Family members’ responses to having a second child	Firstborn’s attitude toward having a second child during mother’s pregnancy	
	Supportive	0.678
	Not explicitly supportive	0.252
	Not asked due to young age	0.070
	Firstborn’s happiness change after birth of sibling	
	Happier	0.572
	Same	0.315
	Less happy	0.112
	Husband’s attitude toward having a second child during the pregnancy	
	Supportive	0.716
	Not explicitly supportive	0.284
	Change in husband’s share of housework before and after having second child	
	Increased	0.302
	No increase	0.698
Factors constraining parenting two children	Work–family conflict, i.e., any negative effect of having second child on one’s career development	
	No negative effect	0.242
	Moderate negative effect	0.421
	Strong negative effect	0.337
	Perceived pressure of parenting two children	
	No pressure	0.290
	Moderate pressure	0.309
	Strong pressure	0.401
Family orientation	Willingness to sacrifice one’s career development to have a second child	
	Very willing	0.218
	Moderately willing	0.347
	Reluctant	0.180
	NA (not professional woman)	0.255

^1^ For each categorical variable (e.g., the response variable), “Statistics” refers to proportions of categories of the variable; for each continuous variable (e.g., mother’s age at the time of survey), “Statistics” refers to mean, with standard deviation included in the bracket. ^2^ Mother’s occupation, timewise inflexible job: job with fixed office hours (according to national occupation classification). ^3^ Family annual income, low income: below 40,000 Chinese yuan a year; middle income: between 40,000 and 100,000 yuan a year; high income: above 100,000 yuan a year. ^4^ Son/son: first and second child were both sons.

**Table 2:** Estimates and relative importance evaluation based on partial ordered logistic regression analysis of mother’s life satisfaction after having a second child ^1^.

Group of Predictors	Predictors	Log-Odds Ratio		ΔAIC_c_^2^
		1|2	2|3	
**Proportional odds effects**				
Background factors	Mother’s occupation (CG: timewise inflexible) ^3^			−4.34
	Timewise flexible	−0.031 (0.969 ^4^)		
	Housewife	−0.342 (0.710)		
	Others	0.031 (1.032)		
	Husband’s occupation (CG: timewise inflexible)			−5.38
	Timewise flexible	−0.075 (0.928)		
	Jobless	0.342 (1.408)		
	Others	−0.356 (0.701)		
	Mother’s education (CG: pre-college level)			−3.09
	College/university level	0.304 (1.356)		
	Postgraduate level	0.538 (1.713)		
	Husband’s education (CG: pre-college level)			−4.4
	College/university level	0.135 (1.145)		
	Postgraduate level	0.222 (1.248)		
	Annual family income (CG: low level)			−2.8
	Middle level	−0.278 (0.757)		
	High level	−0.066 (0.937)		
	Family settlement (CG: main city)			−3.78
	Inner suburb	−0.237 (0.789)		
	Outer suburb	−0.148 (0.863)		
	Life course since second birth (months)	0.017 (1.017)		−4.02
	Quadratic term of life course since second birth (months)	0.003 (1.003)		
	Firstborn child’s age	0.017 (1.017)		−2.23
Fertility preference	Desire to have a second child before the birth of the child	−0.404 (0.668) **		8.33
	How was the second birth planned? (CG: planned pregnancy)			−0.22
	Mistimed pregnancy	−0.157 (0.855)		
	Unwanted pregnancy	−0.497 (0.608) *		
	Sex composition of children (CG: son/son ^5^)			5.01
	Son/daughter	0.795 (2.215) ***		
	Daughter/son	0.545 (1.725) *		
	Daughter/daughter	0.425 (1.529) ^†^		
Family members’ responses to having a second child	Firstborn child’s attitude toward having a sibling during the mother’s pregnancy (CG: supportive)			−2.36
	Not explicitly supportive	0.325 (1.384)		
	Not asked due to young age	0.121 (1.129)		
	Husband’s attitude toward having a second child during the pregnancy (CG: supportive)			0.82
	Not explicitly supportive	−0.362 (0.696) ^†^		
Family orientation	Willingness to sacrifice career development to parent a second child (CG: very willing)			10.25
	Moderately willing	−0.898 (0.408) ***		
	Reluctant	−1.044 (0.352) ***		
	NA (not professional woman)	−0.657 (0.518) *		
**Non-proportional odds effects**				
Background factors	Mother’s age	0.088 (1.091) *^,6^	−0.006 (0.995) ^7^	2.79
Postnatal depression	Did you ever feel depressed during the first month after the second birth? (CG: yes)			6.16
	No	−0.849 (0.428) **	−0.042 (0.959)	
Responses of family members	Firstborn’s happiness after birth of the second child (CG: happier)			3.51
	Same	0.373 (1.453)	0.583 (1.791) *	
	Less happy	0.816 (2.262) *	−0.029 (0.971)	
	Change in husband’s share of housework after the second child’s birth (CG: increased)			7.58
	No increase	−0.293 (0.746)	0.578 (1.783) **	
Factors constraining parenting two children	Work–family conflict, i.e., effect of having a second child on career development (CG: no negative effect)			7.96
	Moderate negative effect	0.641 (1.898) ^†^	0.935 (2.547) ***	
	Strong negative effect	0.942 (2.565) **	0.666 (1.947) *	
	Pressure of parenting two children (CG: no pressure)			24.41
	Moderate pressure	0.154 (1.167)	−0.517 (0.596) *	
	Strong pressure	1.358 (3.890) ***	−0.007 (0.993)	
Intercepts	Intercepts	−6.213 ***^,8^	−0.868 ^9^	
*χ* ^2^		96.98 ***^,10^		
*Df*		49		

^1^ The following predictors did not satisfy the proportional odds assumption, so there were two coefficients for each of their regressors (these coefficients were coded as *θ* in the model): mother’s age, postnatal depression, firstborn’s happiness after a sibling’s birth, change in husband’s share of housework, work–family conflict, pressure of parenting two children. The regressors of other predictors had only one coefficient, i.e., a regressor’s coefficient was the same in predicting logit[*P*(*Y* ≤ 1)] and logit[*P*(*Y* ≤ 2)] (these coefficients were coded as *β* in the model). Here, 1: less life satisfaction after having a second child; 2: same satisfaction; 3: more satisfied; 1|2: cutoff corresponding to logit[*P*(*Y* ≤ 1)]; 2|3: cutoff corresponding to logit[*P*(*Y* ≤ 2)]. ^2^ ΔAIC_c_ = AIC_c,simplified_ − AIC_c,base_ (simplified model was obtained by removing predictors in question from the base model). ^3^ CG, comparison group. ^4^ Numbers in parentheses refer to odds ratio corresponding to log-odds ratio. ^5^ Son/son: first and second child were both sons. ^6^ Coefficient of mother’s age in predicting *P*(*Y* ≤ 1). ^7^ Coefficient of mother’s age in predicting *P*(*Y* ≤ 2). ^8^ First intercept for regression model, i.e., in predicting *P*(*Y* ≤ 1). ^9^ Second intercept for regression model, i.e., in predicting *P*(*Y* ≤ 2). ^10^ The log-likelihood of the model was −518.99 and of the corresponding null model was −615.97. ^†^ *p* < 0.10; * *p* < 0.05; ** *p* < 0.01; *** *p* < 0.001.
